# Inactivation of *Bacillus cereus* vegetative cells by gastric acid and bile during *in vitro* gastrointestinal transit

**DOI:** 10.1186/1757-4749-4-11

**Published:** 2012-10-03

**Authors:** Siele Ceuppens, Mieke Uyttendaele, Stefanie Hamelink, Nico Boon, Tom Van de Wiele

**Affiliations:** 1 Faculty of Bioscience Engineering, Laboratory of Food Microbiology and Food Preservation (LFMFP), Ghent University, Ghent, Belgium; 2 Faculty of Bioscience Engineering, Laboratory of Microbial Ecology and Technology (LabMET), Ghent University, Coupure Links 653, B-9000, Gent, Belgium

**Keywords:** *Bacillus cereus*, Bile, *In vitro* simulation, Gastrointestinal passage

## Abstract

**Background:**

The foodborne pathogen *Bacillus cereus* can cause diarrhoeal food poisoning by production of enterotoxins in the small intestine. The prerequisite for diarrhoeal disease is thus survival during gastrointestinal passage.

**Methods:**

Vegetative cells of 3 different *B. cereus* strains were cultivated in a real composite food matrix, lasagne verde, and their survival during subsequent simulation of gastrointestinal passage was assessed using *in vitro* experiments simulating transit through the human upper gastrointestinal tract (from mouth to small intestine).

**Results:**

No survival of vegetative cells was observed, despite the high inoculum levels of 7.0 to 8.0 log CFU/g and the presence of various potentially protective food components. Significant fractions (approx. 10% of the consumed inoculum) of *B. cereus* vegetative cells survived gastric passage, but they were subsequently inactivated by bile exposure in weakly acidic intestinal medium (pH 5.0). In contrast, the low numbers of spores present (up to 4.0 log spores/g) showed excellent survival and remained viable spores throughout the gastrointestinal passage simulation.

**Conclusion:**

Vegetative cells are inactivated by gastric acid and bile during gastrointestinal passage, while spores are resistant and survive. Therefore, the physiological form (vegetative cells or spores) of the *B. cereus* consumed determines the subsequent gastrointestinal survival and thus the infective dose, which is expected to be much lower for spores than vegetative cells. No significant differences in gastrointestinal survival ability was found among the different strains. However, considerable strain variability was observed in sporulation tendency during growth in laboratory medium and food, which has important implications for the gastrointestinal survival potential of the different *B. cereus* strains.

## Background

*B. cereus* can cause emetic and diarrhoeal and food poisoning by production of resp. emetic (cereulide) and diarrhoeal toxins (non-haemolytic enterotoxin (Nhe), haemolysin BL (Hbl), cytotoxin K (CytK), etc.) [1]. In contrast to the extremely stable toxin cereulide, the enterotoxins are easily degraded by acid and digestive enzymes (proteases) and thus preformed enterotoxins in food do not retain their toxicity during gastrointestinal passage [2]. Therefore, the prerequisite for diarrhoeal food poisoning is enterotoxin production by *B. cereus* in the small intestine, so the gastrointestinal survival of vegetative *B. cereus* was investigated. It was previously shown that approx. 10% of the vegetative cells survived gastric passage [3]. Next, the surviving bacteria are confronted with bile in the lumen of the duodenum, the proximal part of the small intestine.

Bile mainly consists of bile acids (approximately 72% of the total lipids), besides phospholipids (approx. 24%) and cholesterol (approx. 4%) [4]. In humans, these bile acids consist mainly of cholic acid (between 50% and 80%) and chenodeoxycholic acid (between 20% and 50%) [5,6]. They are synthesized in the liver from cholesterol and conjugated to glycine (approx. 75%) or taurine (approx. 25%), with conjugation ratios depending on the diet amongst other factors [6,7]. Secretion of bile is triggered by fat and acid release from the stomach into the duodenum, which results in 7 to 15 mM bile salts in the small intestine after a meal, corresponding with 5 to 10 g Oxgall/L [7-10]. Deconjugation of bile acids by indigenous intestinal bacteria mainly occurs in the distal ileum, where approx. 95% of the bile acids is reabsorbed, of which approx. 15% is unconjugated [7,10]. Both conjugated and unconjugated bile acids are absorbed by passive diffusion along the entire gut, but this process is more efficient for unconjugated bile acids. Additionally, specific active transport systems are present in the distal ileum, which are more efficient in the uptake of conjugated bile acids. After absorption from the intestine, the bile acids are transported to the liver via the blood, reconjugated and secreted again into the bile bladder. This recycling process of bile acids is called enterohepatic circulation.

The physiological role of bile acids is to increase the solubility of dietary fat and facilitate its degradation and absorption. Due to their detergent properties, bile acids also alter cell membranes and thus have cytotoxic and bactericidal effects, noticeable by an increased membrane fluidity and permeability [11-14]. Depending on the bile concentration, disruption of the cell membrane integrity occurs nearly instantaneously, causing cell leakage and death, or more slowly and subtly by altering the membrane permeability and fluidity, the activity of critical proteins in the cell membrane and the membrane hydrophobicity [15]. In addition to destruction of the bacterial cell membrane integrity, bile also induces DNA damage, denaturation and misfolding of proteins, leading to the death of bacteria [16].

Gram-positive bacteria tend to be more sensitive to bile than Gram-negative bacteria, but bile tolerance is very strain specific, so generalized statements for species are not possible [17-19]. Despite the bactericidal effects of bile, some micro-organisms have developed bile resistance by induction of bile metabolizing enzymes and transport systems or by altering membrane permeability, fluidity or charge. Some enteric pathogens may even depend on bile as a host signal for virulence regulation. For example, Salmonella enterica serovar Typhimurium possesses the multidrug efflux pump AcrAB for bile removal and transport through the outer membrane [20]. Moreover, invasion of the host’s epithelial cells by this bacterium is induced by lowered bile concentrations, so after transit to the distal ileum or into the mucus layer [21].

The co-ingestion of food is an important factor in the antibacterial activity of bile in the intestines. Bile inactivation of bacteria is influenced by the presence of food components, which can create protective micro-environments or bind bile. For example, the maximum bile concentration tolerated by *B. cereus* during growth in intestinal medium was 3 g/L porcine bile when pea soup was supplemented, while only 0.9 g/L and 0.6 g/L bile were tolerated in the presence of milk and the absence of food, respectively [22]. Also for Lactobacillus curvatus, the presence of a food matrix, namely meat, increased its bile tolerance and subsequently its gastrointestinal survival [23]. Similarly, the bile tolerance of Bifidobacterium breve was enhanced by soy proteins [24].

In this study, the gastrointestinal survival of vegetative *B. cereus* cells was investigated and linked to the bactericidal role of bile. The *B. cereus* inoculum was cultivated in the composite food matrix lasagne verde prior to the *in vitro* simulation of the gastrointestinal passage to include any potential protective effects of the food particles on the gastrointestinal survival of *B. cereus*.

## Materials and methods

### *B. cereus* strains, cultivation and enumeration

The *B. cereus* strains ATCC 14579, NVH 1230–88 and FF 73 (Table 1) were cultivated and subsequently subcultured in 10 mL Tryptone Soya Broth (TSB, Oxoid) for 24 h at 30°C. After centrifugation and resuspension in 1 mL Physiological Peptone Salt solution (PPS, 8.5 g/L NaCl (Fluka) and 1 g/L neutralized bacteriological peptone (Oxoid)), 830 μL of the subculture was inoculated on 83 g lasagne verde (purchased in the local supermarket) in stomacher bags and incubated for 24 h at 22°C. Retail lasagne verde was purchased in the local supermarket, which contained on average 4.7 log CFU/g total bacteria (standard deviation of 1.3 log CFU/g, analysis of 11 different products during a 6 month period) and ≤ 2.0 log CFU/g *B. cereus*. The average pH of this food product was 5.52 (standard deviation ± 0.06).

**Table 1:** **The simulation of gastrointestinal passage was performed with three different *B. cereus *strains: the type strain *B. cereus *ATCC 14579, the clinical isolate *B. cereus *NVH 1230–88 from a diarrhoeal food poisoning outbreak and the food isolate *B. cereus *FF 73 from the relevant food matrix, namely lasagne verde**

***B. cereus*strain**	**Origin**	**Mininal growth temperature (°C)**	**Hbl production**	**Nhe production**
ATCC^1^14579	type strain	>10	+	+
NVH^2^ 1230-88	clinical (human faeces)	8	+	-
FF^3^ 73	food (lasagne verde)	10	+	+

^1^ATCC = American Type Culture Collection, USA; ^2^NVH = Norwegian School of Veterinary Science, Oslo, Norway; ^3^FF = Flanders’ Food Collection, Belgium.

The total count and *B. cereus* count were determined by plating the appropriate dilutions in PPS on Tryptone Soya Agar (TSA) and Mannitol-Egg yolk-Polymyxin B agar (MYP, respectively. Spore concentrations were determined by plating after heating at 80°C for 10 min.

### *In vitro* simulation of gastrointestinal passage

The dynamic gastrointestinal simulation experiment comprised five phases: 1) the mouth, 2) the stomach, 3) the duodenum, 4) dialysis and 5) the ileum [25]. Briefly, the lasagne verde containing the *B. cereus* inoculum (83 g) was mixed with saliva medium (56 mL, pH 6.5, 37°C) by stomaching for 1 min (Stomacher Lab Blender 400, Seward) and incubated for 10 min before transfer to the stomach vessel. The gastric pH was decreased from 5.0 to 3.0 during the first 90 min and to 2.0 during the last 90 min by continuously added acid (0.28 M HCl). Gastric emptying was initiated 30 min after the start of the gastric phase in 5 fractions by discontinuous pumping in such a way that approx. 25% of the gastric content was removed after 1 h, 50% after 2 h and 75% after 3 h. The fractional gastric emptying resulted in a 150 min overlap between the stomach and duodenum phase, in which the *B. cereus* inoculum was divided in subpopulations which were subjected to various different incubation times in the stomach phase (min. 30 min, max. 180 min) and duodenum phase (min. 10 min, max. 160 min). The intestinal vessel was anaerobic (flushed with nitrogen gas) and contained intestinal medium with 10.0 g/l bile (Oxgall, Difco) and the pH pH was automatically adjusted by a pH controller (FerMac 260, Electrolab) to remain at pH 5.0 during the first 45 min and at pH 6.0 during the last 115 min. And the end of the duodenum phase, the complete gastric contents were transfered and the bile concentration was lowered by dilution to 5 g/L. During the next phase, ≥ 90% of this bile was removed by dialysis and competition with human intestinal bacteria was simulated during the final ileum phase. However, after elimination of the *B. cereus* vegetative cells, the gastrointestinal experiment was stopped, resulting in experiments consisting of the first three phases only. The experiments were performed in triplicate with different *B. cereus* inocula on different days.

### Bile tolerance of *B. cereus*

*B. cereus* strain NVH 1230–88 was cultivated and subsequently subcultured in 10 mL TSB for 24 h at 30°C. Then 100 μL of the subculture was inoculated in glass tubes containing 9.9 mL TSB and incubated at 37°C. The pH of the TSB was either neutral (pH = 7.2) or acidic (pH = 5.0) and supplemented with varying bile concentrations, resulting in the final concentrations of 0.0 (TSB control), 1.0, 5.0 and 10.0 g/L oxgall (Difco). The survival of vegetative *B. cereus* cells was assessed by plating the appropriate diltutions in PPS on TSA.

### Toxin production

Production of enterotoxins Nhe and Hbl was determined by analyzing 1 mL samples after filtration (0,2 μm syringe filters, Whatman) with the Duopath® Cereus Enterotoxins (Merck) according to the manufacturers’ instructions.

## Results

### Growth of *B. cereus* in lasagne verde

The lasagne verde was inoculated with approx. 6.0 CFU/g *B. cereus* and incubated for 24 h at 22°C to obtain a highly concentrated inoculum of vegetative *B. cereus* cells grown in a composite food matrix with food microbiota by simulating storage of contaminated lasagne at room temperature. This resulted in lasagne verde containing approx. 8.5 log CFU/g total bacteria, of which *B. cereus* constituted 7.0 to 8.0 CFU/g, depending on the strain (Table 2). *B. cereus* NVH 1230–88 showed a stable spore concentration of 3.5 log spores/g, originating from the TSB inoculum. In contrast, additional sporulation was apparent from approx. 4.0 log spores/g to 4.5 log spores/g after 30 h for *B. cereus* FF 73. Remarkably, *B. cereus* ATCC 14579 did not produce any spores after 30 h incubation, with only very low numbers (3.0 spores/g) observed in only 1 of the 3 replicate bags. After inoculation and incubation, the lasagne verde was thus highly contaminated with *B. cereus* vegetative cells (7.0 to 8.0 log CFU/g) in the stationary growth phase and low amounts of *B. cereus* spores (up to 4.0 log spores/g).

**Table 2:** **Growth of the three different *B. cereus *strains in the food matrix prior to simulation of gastrointestinal passage; average values of independent experiments in triplicate expressed in log CFU/g ± standard deviation are presented**

***B. cereus *strain**	**Time (h)**					
	**0**	**22**	**24**	**26**	**28**	**30**
*B. cereus* ATCC14579						
Total count	5.8 ± 0.1	8.3 ± 0.6	8.4 ± 0.3	8.8 ± 0.2	8.8 ± 0.3	8.9 ± 0.2
Total *B. cereus*	5.7 ± 0.1	6.9 ± 0.1	6.9 ± 0.1	6.7 ± 0.3	7.2 ± 0.3	7.0 ± 0.2
*B. cereus* spores	< 2.0 ± 0.0	< 2.0 ± 0.0	< 2.0 ± 0.0	< 2.0 ± 0.0	< 2.0 ± 0.0	2.3 ± 0.6
*B. cereus* NVH 1230-88						
Total count	5.9 ± 0.1	8.2 ± 0.2	8.4 ± 0.1	8.9 ± 0.0	8.9 ± 0.1	8.8 ± 0.2
Total *B. cereus*	6.2 ± 0.3	7.9 ± 0.2	8.2 ± 0.1	8.5 ± 0.2	8.3 ± 0.3	7.9 ± 0.1
*B. cereus* spores	3.5 ± 0.6	3.5 ± 0.2	3.3 ± 0.6	3.4 ± 0.6	3.3 ± 0.6	3.4 ± 0.6
*B. cereus* FF 73						
Total count	5.7 ± 0.1	8.5 ± 0.1	8.7 ± 0.1	9.0 ± 0.0	9.0 ± 0.3	8.9 ± 0.3
Total *B. cereus*	5.7 ± 0.1	8.1 ± 0.2	8.0 ± 0.1	8.3 ± 0.1	8.2 ± 0.1	8.3 ± 0.2
*B. cereus* spores	3.8 ± 0.2	3.9 ± 0.4	3.9 ± 0.4	4.0 ± 0.5	4.0 ± 0.3	4.5 ± 0.1

No production of enterotoxins was detected in lasagne verde after 24 h by *B. cereus* ATCC 14579 and *B. cereus* NVH 1230–88, while Nhe was sporadically detected (in 1 out of 3 replicates) in the case of *B. cereus* FF 73. This was probably due to the acidic pH 5.5 of lasagne verde and the rather low incubation temperature of 22°C, which are not optimal for enterotoxin production [1]. However, preformed enterotoxins in food are not responsible for the diarrhoeal food poisoning, since they are rapidly degraded during gastrointestinal passage [2].

### *In vitro* simulation of gastrointestinal passage of *B. cereus* vegetative cells

**Figure 1:**
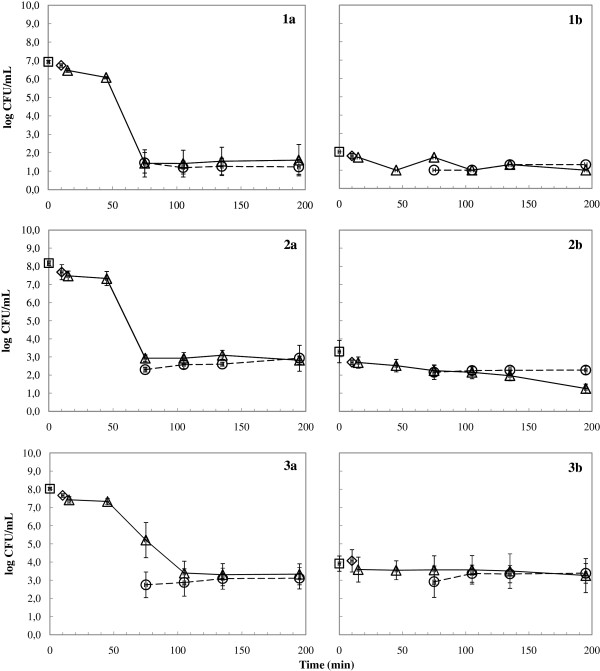
**Inactivation of vegetative cells during the dynamic *in vitro *simulation of gastrointestinal passage with *B. cereus *ATCC 14579** (**1**), *B. cereus* NVH 1230–88 (**2**) and *B. cereus* FF 73 (**3**); the *B. cereus* total (**a**) and spore (**b**) counts were determined by plating on MYP without and with heat treatment (10 min at 80°C), respectively, in the lasagne verde (□), the mouth phase (◊), the stomach phase (Δ) and the duodenum phase (○); the average values and standard deviation of triplicate experiments on different days are presented.

The lasagne verde contaminated with 7.0 to 8.0 log CFU/mL *B. cereus* vegetative cells in the stationary growth phase was subjected to *in vitro* simulation of gastrointestinal passage (Figure 1). Survival of *B. cereus* (< 1 log reduction) was observed during the mouth phase and the first 30 min of the stomach phase when the gastric pH was between 5.0 and 4.0. Thereafter, when the gastric pH decreased below 4.0, the vegetative cells were rapidly inactivated in the stomach phase, noticeable as a rapid decline of the total *B. cereus* counts to similar values of the *B. cereus* spore counts in the gastric vessels. The *B. cereus* total and spore counts during the duodenum phase remained approx. 1.0 log CFU/mL for *B. cereus* ATCC 14579, approx. 2.5 log CFU/mL for *B. cereus* NVH 1230–88 and approx. 3.5 log CFU/mL for *B. cereus* FF 73. Interestingly, the spore counts of *B. cereus* NVH 1230–88 decreased during the stomach phase during the last hour and most acidic pH. Since the total *B. cereus* count did not change, this indicates initiation of spore germination. *B. cereus* ATCC 14579 showed inconsistent spore production, resulting in the absence of spores during the first two simulations and very low numbers (slightly above the detection limit of 1.0 spores/mL) during the last two experiments.

No detection tests for enterotoxins Nhe and Hbl were performed, since no growth was observed during any of the gastrointestinal phases, which is a prerequisite for enterotoxin production [1].

### Bile tolerance of *B. cereus*

**Figure 2:**
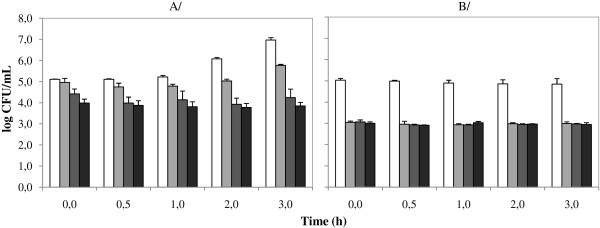
**Survival of *B. cereus *NVH 1230–88 in Tryptone Soya Broth (TSB) at 37°C with pH 7.2 (A) and pH 5.0 (B) with varying bile (Oxgall, Difco) concentrations: 0.0 g/L (white), 1.0 g/L (light grey), 5.0 g/L (dark grey) and 10.0 g/L (black).**

The growth and survival of *B. cereus* NVH 1230–88 was determined in the presence of varying bile concentrations (Figure 2). At optimal conditions, i.e. neutral TSB with pH 7.2, the effect of bile was concentration dependent. The lowest concentration of 1.0 g/L Oxgall resulted in a population reduction of approx. 30% in comparison with the control (TSB without bile) at time 0 h. Moreover, after 1 h the viable count was reduced with approx. 40% in comparison with the concentration in TSB with 1.0 g/L Oxgall at 0 h. However, after 2 h recovery and outgrowth of the remaining cells was observed, because the *B. cereus* concentration was similar to that of 0 h, and finally after 3 h, the population had increased with approx. 1 log in comparison with that at 0 h. However, the presence of 5.0 g/L Oxgall induced a permanent decline to 4.1 log CFU/mL on average (inactivation of 88% of the vegetative cells) and that of 10.0 g/L Oxgall to 3.9 log CFU/mL (inactivation of 94%).

In TSB at acidic pH 5.0, approx. 99% of the *B. cereus* inoculum was instantly inactivated by the bile exposure, regardless of the bile concentration, resulting in constant total *B. cereus* counts of approx. 3.0 log CFU/mL. The *B. cereus* inoculum contained between 0.1 and 0.2% spores, so constant spore counts were obtained throughout the experiment: 2.6 (± 0.2) log spores/mL at the beginning and 2.5 (± 0.3) log spores/mL at the end. Taking this spore population into account, the vegetative cells still constituted the majority (approx. 60%) of the 1% bile resistant *B. cereus*.

## Discussion

The survival of vegetative cells during gastric passage was similar to that of previous studies with mashed potato medium [3]. During those experiments, approx. 10% of the vegetative cells survived gastric passage, most of them in the first gastric fraction when gastric pH was between 5.0 and 4.0. These cells were transferred alive into the intestinal vessel and thus between 5.5 and 6.5 log CFU/mL *B. cereus* were expected in the intestinal vessel. However, much lower counts, which approximated the *B. cereus* spore counts, were obtained during the duodenum phase. This indicates that the vegetative cells which survived gastric passage were inactivated in the intestinal environment. Batch incubation experiments in TSB and varying concentrations of bile showed that the majority (99.1%) of vegetative cells was inactivated immediately after bile exposure at pH 5.0, regardless of the bile concentration in the range of 1.0 to 10.0 g Oxgall/L. In conclusion, vegetative *B. cereus* cells which survived gastric passage were eliminated during the subsequent duodenum phase by the bactericidal effect of bile.

The bactericidal effect of bile on vegetative *B. cereus* cells was pH dependent. At pH 7.0, the inactivation was correlated with the bile concentration, and the lowest bile concentration (1 g/L) even allowed survival and growth. In contrast, at pH 5.0, instant inactivation of the majority (> 99%) of vegetative cells occurred, independent of the bile concentration (in the range of 1.0 to 10.0 g/L). Similar to our results, inactivation of Listeria monocytogenes by bile acids was also strongly increased at acidic pH 5.5 [26]. Inactivation of bacteria by bile is similar to that by organic acids, which are both particularly effective at low pH. The logarithmic acid dissociation constant (pKa) of unconjugated bile acids is approximately 5.1, but conjugation with glycine lowers the pKa to approx. 3.7 [27]. Conjugation with taurine leads to an even lower pKa of approx. 1.5 [28]. As a consequence, the majority of conjugated bile acids is dissociated and ionized at most physiological and intestinal pH values, namely 95.24% at pH 5.0 and 99.95% at pH 7.0 for glycoconjugated bile acids. Only the unionized forms of bile acids can passively cross the cell membrane, while the dissociated and ionized conjugated bile acids require specific active transport systems [29]. As a result, 4.67% of the conjugated bile acids can enter the *B. cereus* cells at pH 5.0, in contrast to only 0.03% at pH 7.0. Moreover, the internal pH of viable *B. cereus* cells always lies between 6.0 and 7.5, depending on the external pH and culture conditions [30]. After the undissociated bile acids have diffused into the *B. cereus* cells, the majority of them (99.50 to 99.98%) undergoes intracellular dissociation, resulting in lethal internal acidification.

It was reported that certain food components increased the bile tolerance of *B. cereus* vegetative cells in intestinal medium with pH 6.5 [22]. However, the composite food matrix lasagne verde did not mitigate the detrimental effects of bile on *B. cereus* during our experiments at pH 5.0. It is possible that food type effects are only observed at a more optimal pH for this foodborne pathogen, when more pronounced differences between treatments are expected.

In contrast to vegetative cells, *B. cereus* spores were not inactivated by bile, although they did not germinate in the intestinal medium containing 5 to 10 g/L Oxgall. This coincides with the observations of the dynamic simulation of gastrointestinal passage of spores [25], where spores were unaffected during the mouth, stomach and duodenum phase and only started germination during the dialysis, when the bile concentration was lowered from 5.0 g/L Oxgall to < 0.5 g/L and those of the batch incubation experiments [31], where spores germinated within 2 h in intestinal simulation medium containing 1.0 g/L bile. Other studies also reported that spores were able to germinate and multiply in simulated intestinal fluid containing bile (1.5 g Oxgall/L) [32] and that the bile resistance of *B. cereus* spores was superior to that of vegetative cells [33]. In conclusion, high bile concentrations (≥ 5 g/L Oxgall) do not allow spore germination, while low bile concentrations (< 5 g/L) induce germination and outgrowth.

Besides its importance in nutrient digestion and absorption, bile is also an important antimicrobial compound in the intestine [7]. Abnormal low intestinal bile levels may occur in people with biliary or liver abnormalities or with deficiencies in the enterohepatic circulation, which may render those people especially susceptible to diarrhoea because of enhanced survival of ingested and intestinal bacteria. Moreover, bile acids are endogenous laxatives, so malabsorption of bile acids from the small intestine, leading to increase bile levels in the terminal small intestine and the colon, may also result in diarrhoea [34].

## Conclusion

*B. cereus* vegetative cells were unable to survive gastrointestinal passage as simulated by the *in vitro* experiment, despite the high inoculum concentration of 7.0 to 8.0 log CFU/mL and the cultivation of the inoculum in the composite food matrix lasagne verde. The majority of vegetative cells was inactivated by gastric acid, and the surviving cells were subsequently inactivated by bile during the duodenum phase. In contrast, *B. cereus* spores survived the simulation of gastrointestinal passage and remained spores during unfavourable conditions for germination (and outgrowth). Therefore, the infective dose for *B. cereus* probably varies according to the physiological form consumed, being lower for the highly resistant spores. Although the gastrointestinal survival was similar for the different strains, their sporulation tendency varied considerably, which in turn has important implications for the gastrointestinal survival potential and thus the infective dose of the different *B. cereus* strains.

## Competing interest

We, all authors, declare that we have no competing interests.

## Authors’ contributions

SC, MU and TVdW designed the experiments; SC and SH performed the experimental work and processed the results; SC wrote the manuscript aided by MU, NB and TVdW. All authors read and approved the final manuscript.

## References

[B1] Ceuppens S Rajkovic A Heyndrickx M Tsilia V Van de Wiele T Boon N Uyttendaele M Regulation of toxin production by *Bacillus cereus* and its food safety implications Crit Rev Microbiol 2011 37 188 213 10.3109/1040841X.2011.558832 21417966

[B2] Ceuppens S Rajkovic A Hamelink S Van de Wiele T Boon N Uyttendaele M Enterotoxin production by *Bacillus cereus* under gastrointestinal conditions and their immunological detection by commercially available kits Foodborne Pathog Dis 2012 in press 10.1089/fpd.2012.1230 23237409

[B3] Ceuppens S Uyttendaele M Drieskens K Rajkovic A Boon N Van de Wiele T Survival of *Bacillus cereus* vegetative cells and spores during *in vitro* simulation of gastric passage J Food Prot 2012 75 690 694 10.4315/0362-028X.JFP-11-481 22488056

[B4] Hay DW Carey MC Chemical species of lipids in bile Hepatology 1990 12 S6 S12 2210659

[B5] Ellis E Goodwin B Abrahamsson A Liddle C Mode A Rudling M Bjorkhem I Einarsson C Bile acid synthesis in primary cultures of rat and human hepatocytes Hepatology 1998 27 615 620 10.1002/hep.510270241 9462665

[B6] Sjovall J Dietary lycine and taurine on bile acid conjugation in man - Bile acids and steroids Proc Soc Exp Biol Med 1959 100 676 678 13645682 10.3181/00379727-100-24741

[B7] Begley M Gahan CG Hill C The interaction between bacteria and bile FEMS Microbiol Rev 2005 29 625 651 10.1016/j.femsre.2004.09.003 16102595

[B8] Hagens WI Lijzen JPA Sips AJAM Oomen AG Richtlijn: bepalen van de orale biobeschikbaarheid van lood in de bodem RIVM rapport 711701060/2007 2007 63p

[B9] Minekus M Marteau P Havenaar R Huis in 't Veld JHJ A multicompartmental dynamic computer controlled model simulating the stomach and small intestine ATLA Altern Lab Anim 1995 23 197 209

[B10] Northfield TC Mccoll I Postprandial concentrations of free and conjugated bile acids down length of normal human small intestine Gut 1973 14 513 518 10.1136/gut.14.7.513 4729918 PMC1412809

[B11] Palmeira CM Rolo AP Mitochondrially-mediated toxicity of bile acids Toxicology 2004 203 1 15 10.1016/j.tox.2004.06.001 15363577

[B12] Pazzi P Puviani AC Dalla LM Guerra G Ricci D Gullini S Ottolenghi C Bile salt-induced cytotoxicity and ursodeoxycholate cytoprotection: *in vitro* study in perifused rat hepatocytes Eur J Gastroenterol Hepatol 1997 9 703 709 10.1097/00042737-199707000-00011 9262981

[B13] Schroder O Rathner W Caspary WF Stein J Bile acid-induced increase of rat colonic apical membrane fluidity and proton permeability Z Gastroenterol 1996 34 365 370 8767825

[B14] Zhao DL Hirst BH Bile salt-induced increases in duodenal brush-border membrane proton permeability, fluidity, and fragility Dig Dis Sci 1990 35 589 595 10.1007/BF01540406 2158881

[B15] Noh DO Gilliland SE Influence of bile on cellular integrity and beta-galactosidase activity of Lactobacillus acidophilus J Dairy Sci 1993 76 1253 1259 10.3168/jds.S0022-0302(93)77454-8 8505417

[B16] Flahaut S Frere J Boutibonnes P Auffray Y Comparison of the bile salts and sodium dodecyl sulfate stress responses in Enterococcus faecalis Appl Environ Microbiol 1996 62 2416 2420 8779581 10.1128/aem.62.7.2416-2420.1996 PMC168024

[B17] Chateau N Castellanos I Deschamps AM Distribution of pathogen inhibition in the Lactobacillus isolates of a commercial probiotic consortium J Appl Bacteriol 1993 74 36 40 10.1111/j.1365-2672.1993.tb02993.x 8420917

[B18] Hyronimus B Le MC Sassi AH Deschamps A Acid and bile tolerance of spore-forming lactic acid bacteria Int J Food Microbiol 2000 61 193 197 10.1016/S0168-1605(00)00366-4 11078170

[B19] Margolles A Garcia L Sanchez B Gueimonde M de los Reyes-Gavilan CG Characterisation of a Bifidobacterium strain with acquired resistance to cholate – a preliminary study Int J Food Microbiol 2003 82 191 198 10.1016/S0168-1605(02)00261-1 12568759

[B20] Nikaido H Basina M Nguyen V Rosenberg EY Multidrug efflux pump AcrAB of Salmonella Typhimurium excretes only those beta-lactam antibiotics containing lipophilic side chains J Bacteriol 1998 180 4686 4692 9721312 10.1128/jb.180.17.4686-4692.1998 PMC107484

[B21] Prouty AM Gunn JS Salmonella enterica serovar typhimurium invasion is repressed in the presence of bile Infect Immun 2000 68 6763 6769 10.1128/IAI.68.12.6763-6769.2000 11083793 PMC97778

[B22] Clavel T Carlin F Dargaignaratz C Lairon D Nguyen-The C Schmitt P Effects of porcine bile on survival of *Bacillus cereus* vegetative cells and Haemolysin BL enterotoxin production in reconstituted human small intestine media J Appl Microbiol 2007 103 1568 1575 10.1111/j.1365-2672.2007.03410.x 17953568

[B23] Ganzle MG Hertel C van der Vossen JMBM Hammes WP Effect of bacteriocin-producing lactobacilli on the survival of Escherichia coli and Listeria in a dynamic model of the stomach and the small intestine Int J Food Microbiol 1999 48 21 35 10.1016/S0168-1605(99)00025-2 10375132

[B24] Shimakawa Y Matsubara S Yuki N Ikeda M Ishikawa F Evaluation of Bifidobacterium breve strain Yakult-fermented soymilk as a probiotic food Int J Food Microbiol 2003 81 131 136 10.1016/S0168-1605(02)00224-6 12457587

[B25] Ceuppens S Uyttendaele M Drieskens K Heyndrickx M Rajkovic A Boon N Van de Wiele T Survival and germination of *Bacillus cereus* spores during *in vitro* simulation of gastrointestinal transit occurred without outgrowth and enterotoxin production Appl Environ Microbiol 2012 in press 10.1128/AEM.02142-12 PMC3485738 22923409

[B26] Begley M Gahan CGM Hill C Bile stress response in Listeria monocytogenes LO28: adaptation, cross-protection, and identification of genetic loci involved in bile resistance Appl Environ Microbiol 2002 68 6005 6012 10.1128/AEM.68.12.6005-6012.2002 12450822 PMC134417

[B27] Hofmann AF Mysels KJ Bile acid solubility and precipitation *in vitro* and in vivo - the role of conjugation, pH, and Ca2+ ions J Lipid Res 1992 33 617 626 1619357

[B28] Goto J Suzaki K Chikai T Nagase K Nambara T Studies on steroids: Separation of bile acid 3-glucuronides by High-Performance Liquid-Chromatography J Chromatogr 1985 348 151 157 4086635 10.1016/s0021-9673(01)92448-3

[B29] St-Pierre MV Kullak-Ublick GA Hagenbuch B Meier PJ Transport of bile acids in hepatic and non-hepatic tissues J Exp Biol 2001 204 1673 1686 11316487 10.1242/jeb.204.10.1673

[B30] Senouci-Rezkallah K Schmitt P Jobin MP Amino acids improve acid tolerance and internal pH maintenance in *Bacillus cereus* ATCC14579 strain Food Microbiol 2011 28 364 372 10.1016/j.fm.2010.09.003 21356439

[B31] Ceuppens S Van de Wiele T Rajkovic A Ferrer-Cabaceran T Heyndrickx M Boon N Uyttendaele M Impact of intestinal microbiota and gastrointestinal conditions on the *in vitro* survival and growth of *Bacillus cereus* Int J Food Microbiol 2012 155 241 246 10.1016/j.ijfoodmicro.2012.02.013 22436640

[B32] Wijnands LM Dufrenne JB Zwietering MH van Leusden FM Spores from mesophilic *Bacillus cereus* strains germinate better and grow faster in simulated gastro-intestinal conditions than spores from psychrotrophic strains Int J Food Microbiol 2006 112 120 128 10.1016/j.ijfoodmicro.2006.06.015 16860423

[B33] Kristoffersen SM Ravnum S Tourasse NJ Okstad OA Kolsto AB Davies W Low concentrations of bile salts induce stress responses and reduce motility in *Bacillus cereus* ATCC 14579 J Bacteriol 2007 189 5302 5313 10.1128/JB.00239-07 17496091 PMC1951874

[B34] Potter GD Bile acid diarrhea Dig Dis 1998 16 118 124 10.1159/000016855 9571377

